# Supplementary material for: FPocketWeb: protein pocket hunting in a web browser
Source: J Cheminform. 2022 Aug 26;14:58. doi: 10.1186/s13321-022-00637-0 (PMC9414105; doi:10.1186/s13321-022-00637-0)
Supplement: Supplementary file 1 — Additional file 1. The FPocketWeb source code, version 1.0.1. See http://durrantlab.com/fpocketweb-download for the latest version. [file 13321_2022_637_MOESM1_ESM.zip › fpocketweb-1.0.1/src/UI/Tabs/templates/params.htm]

Use this tab to setup a FPocketWeb job in your browser. Specify the
input files and *fpocket* parameters below.


Use Example File


Optional output parameters


Optional parameters to control FPocketWeb output


Optional input parameters


Advanced parameters that are best left unmodified


Optional pocket detection parameters


Start FPocketWeb

Unfortunately, your browser does not support WebAssembly. Please
switch to a browser that does
(e.g., Google Chrome).

Note that you can still use the "Existing Fpocket Output" option (see menu
on the left) even without WebAssembly.
