# Supplementary figures and images for: FPocketWeb: protein pocket hunting in a web browser
Source: J Cheminform. 2022 Aug 26;14:58. doi: 10.1186/s13321-022-00637-0 (PMC9414105; doi:10.1186/s13321-022-00637-0)

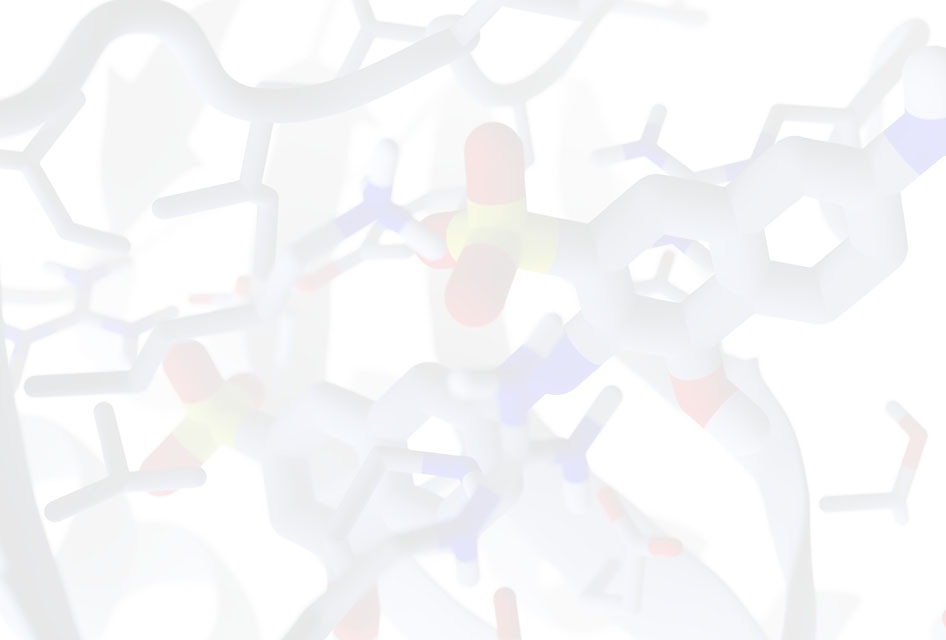

Supplement: Supplementary file 1 — Additional file 1. The FPocketWeb source code, version 1.0.1. See http://durrantlab.com/fpocketweb-download for the latest version. [file 13321_2022_637_MOESM1_ESM.zip › fpocketweb-1.0.1/src/styles/webina_logo.jpg]
